# Supplementary material for: Role and effectiveness of telephone hotlines in outbreak response in Africa: A systematic review and meta-analysis
Source: PLoS One. 2023 Nov 29;18(11):e0292085. doi: 10.1371/journal.pone.0292085 (PMC10686465; doi:10.1371/journal.pone.0292085)
Supplement: S1 File — (DOC) [file pone.0292085.s002.doc]

S1 Table Database search strategy

Database: APA PsycInfo <1995 to September Week 1 2022> , Embase Classic+Embase <1995 to 2022 September 16>, Global Health <1995 to 2022 Week 37>, Ovid MEDLINE(R) ALL <1995 to September 16, 2022>

Search Strategy:

--------------------------------------------------------------------------------

1 hotline.mp. or hotline/ or telephone/ (65188)

2 telephone/ or hotline/ or telephone hotline.mp. (62621)

3 mobile hotline.mp. (2)

4 mobile phone.mp. or mobile phone/ (52970)

5 mobile phone/ or mobile phone line.mp. (39939)

6 toll-free helpline.mp. (44)

7 toll free helpline.mp. (44)

8 alert line.mp. (106)

9 cell phone line.mp. (3)

10 call centre.mp. or call center/ (1451)

11 national hotline.mp. (44)

12 outbreak.mp. or epidemic/ (353677)

13 health emergency.mp. (14461)

14 1 or 2 or 3 or 4 or 5 or 6 or 7 or 8 or 9 or 10 or 11 (117516)

15 12 or 13 (364264)

16 14 and 15 (1428)

17 remove duplicates from 16 (1128)

18 remove duplicates from 16 (1128)

***************************
