# Supplementary material for: Role and effectiveness of telephone hotlines in outbreak response in Africa: A systematic review and meta-analysis
Source: PLoS One. 2023 Nov 29;18(11):e0292085. doi: 10.1371/journal.pone.0292085 (PMC10686465; doi:10.1371/journal.pone.0292085)
Supplement: S2 File — (DOCX) [file pone.0292085.s003.docx]

S2 Table Results of meta-regression using sample size
