# Supplementary material for: Role and effectiveness of telephone hotlines in outbreak response in Africa: A systematic review and meta-analysis
Source: PLoS One. 2023 Nov 29;18(11):e0292085. doi: 10.1371/journal.pone.0292085 (PMC10686465; doi:10.1371/journal.pone.0292085)
Supplement: S4 File — (DOCX) [file pone.0292085.s005.docx]

S4 Table Assessment of the quality of cross-sectional studies

| Item | Alpren et al, 2017 | Lee et al, 2016 | Lopez et al, 2021 | Oladeji al, 2020 | Jalloh et al, 2017 | Keita et al, 2021 | Hemingway-Foday et al,2020 | Coulibaly et al, 2019 |
| --- | --- | --- | --- | --- | --- | --- | --- | --- |
|  |  |  |  |  |  |  |  |  |
| Were the criteria for inclusion in the sample clearly defined? | 1 | 1 | 1 | 1 | 1 | 1 | 1 | 1 |
| Were the study subjects and the setting described in detail? | 0 | 0 | 0 | 0 | 1 | 1 | 1 | 0 |
| Was the exposure measured in a valid and reliable way? | 1 | 1 | 1 | 1 | 1 | 1 | 1 | 1 |
| Were objective, standard criteria used for measurement of the condition? | 1 | 1 | 1 | 1 | 1 | 1 | 1 | 1 |
| Were confounding factors identified? | 0 | 0 | 0 | 0 | 0 | 0 | 0 | 0 |
| Were strategies to deal with confounding factors stated? | 0 | 0 | 0 | 0 | 0 | 0 | 0 | 0 |
| Were the outcomes measured in a valid and reliable way? | 0 | 1 | 1 | 1 | 1 | 1 | 1 | 1 |
| Was appropriate statistical analysis used? | 0 | 0 | 0 | 1 | 1 | 0 | 0 | 0 |
| Score | 4 | 4 | 4 | 5 | 6 | 5 | 5 | 4 |
| Impression | Low quality | Low quality | Poor quality | Moderate quality | Moderate quality | Moderate quality | Moderate quality | Low quality |
