# Supplementary material for: Role and effectiveness of telephone hotlines in outbreak response in Africa: A systematic review and meta-analysis
Source: PLoS One. 2023 Nov 29;18(11):e0292085. doi: 10.1371/journal.pone.0292085 (PMC10686465; doi:10.1371/journal.pone.0292085)
Supplement: S5 File — (DOCX) [file pone.0292085.s006.docx]

S5 Table Assessment of the quality of cohort studies

| Item | Alroy et al,2020 | Gashu et al, 2016 | Oyas et al, 2018 | Kouadio et al,2015 | Rajatorinira et al,2012 | Miller etal,2014 | Jia et al,2015 | Davies et al,2019 |
| --- | --- | --- | --- | --- | --- | --- | --- | --- |
|  |  |  |  |  |  |  |  |  |
| Were the two groups similar and recruited from the same population? | 0 | 0 | 0 | 0 | 0 | 0 | 0 | 0 |
| Were the exposures measured similarly to assign people to both exposed and unexposed groups? | 0 | 0 | 0 | 0 | 0 | 0 | 0 | 0 |
| Was the exposure measured in a valid and reliable way? | 1 | 1 | 1 | 1 | 1 | 1 | 1 | 1 |
| Were confounding factors identified? | 0 | 0 | 0 | 0 | 0 | 0 | 0 | 0 |
| Were strategies to deal with confounding factors stated? | 0 | 0 | 0 | 0 | 0 | 0 | 0 | 0 |
| Were the groups/participants free of the outcome at the start of the study (or at the moment of exposure)? | 0 | 0 | 0 | 0 | 0 | 0 | 0 | 0 |
| Were the outcomes measured in a valid and reliable way? | 1 | 0 | 1 | 1 | 1 | 1 | 1 | 1 |
| Was the follow up time reported and sufficient to be long enough for outcomes to occur? | 1 | 1 | 1 | 1 | 1 | 1 | 1 | 1 |
| Was follow up complete, and if not, were the reasons to loss to follow up described and explored? | 0 | 1 | 0 | 0 | 1 | 1 | 1 | 1 |
| Were strategies to address incomplete follow up utilized? | 0 | 1 | 1 | 0 | 0 | 1 | 0 | 1 |
| Was appropriate statistical analysis used? | 0 | 1 | 0 | 0 | 0 | 0 | 0 | 0 |
| Score | 3 | 5 | 4 | 3 | 4 | 6 | 4 | 5 |
| Impression | Low quality | Low quality | Low quality | Low quality | Low quality | Low quality | Low quality | Low quality |
