# Supplementary material for: Role and effectiveness of telephone hotlines in outbreak response in Africa: A systematic review and meta-analysis
Source: PLoS One. 2023 Nov 29;18(11):e0292085. doi: 10.1371/journal.pone.0292085 (PMC10686465; doi:10.1371/journal.pone.0292085)
Supplement: Attachment — Submitted filename: PONE_review.docx [file pone.0292085.s007.docx]

Thank you for the opportunity to review this paper

The paper is quite well written, below are my comments.

Comments

Abstract

Background:

1. The authors state “In Africa, little is known about the role of telephone hotlines in outbreak response. We systematically reviewed the effectiveness of hotlines on outbreak response in Africa”, little is known about the role and they reviewed the effectiveness. The gap isn’t answered, I would suggest authors say we systematically reviewed the role and effectiveness of hotlines in outbreak response in Africa.
2. Similarly, the title can also be highlighted as Role and effectiveness of telephone hotlines in outbreak response in Africa: a systematic review and meta-analysis

Methods

3. The authors say “during the last 25 years”, It would be good to state the actual period for example from 199x to 202x
4. “The search was also repeated on 16 September 202” please write the year correctly

Results

5. Could the authors also highlight the different outbreaks in which these telephone hotlines were used.

Introduction

6. “Public and private entities have used telephone hotlines to provide general health information and education and have also, in some cases, repurposed them as alert lines for outbreak response.” Please reference
7. “Most reviews of mobile health in low and middle income countries (LMICs) have focused on the use of mobile phones in chronic disease management and health behavior changes in non-outbreak settings (4)” the authors say most reviews but only one citation
8. “This systematic review and meta-analysis assessed the published evidence on the use of telephone hotlines for outbreaks in Africa” the authors need to be consistent; role/ use, or role and effectiveness

Methods

9. There was no restriction on language, were there any papers not in English that were included?
10. Please state the period considered for inclusion of the studies. For example, studies done between this period(xx-yy) were eligible
11. “Two independent individuals (NF and CA) searched citations in MEDLINE, Embase, PsycINFO, and Web of Science.” In the abstract, the authors state that five databases were searched including Global Health, please clarify.

Results

12. Flow chart

1251 records were screened, 1211 were excluded and 41 were assessed for eligibility, this is not adding up

13. “Effect of the hotlines” the authors ought to write this well. Effectiveness of ..
